# Audit of NICE QS101 Learning Disability: Behaviour That Challenges

**DOI:** 10.1192/bjo.2025.10537

**Published:** 2025-06-20

**Authors:** Ambreen Rashid, Bushra Rauf, Rani Pathania, Hafsa Sheikh, Toibat Adeyinka

**Affiliations:** Coventry and Warwickshire Partnership Trust, Coventry, United Kingdom

## Abstract

**Aims:** To check compliance with the NICE guidance for behaviour that challenges, and to identify potential actions/change ideas for areas requiring improvement.

**Methods:** Data collection took place between 15 January and 15 April 2024. Data was collected by clinical staff on proformas based on the NICE guidance, which were co-designed by the Improvement Team and clinical staff. Data was collected using patients’ electronic records held on the Carenotes system and shared drives.

3 pilot proformas were initially completed across 3 different services to assess the robustness of audit proforma and to identify any changes required prior to the main audit. Following the pilot, changes were made to audit proforma after discussion in the audit meeting. Both inpatient and community teams collected data during the above-mentioned timeframe, and data was then sent to the Improvement Team for analysis. Data was input into a Microsoft Excel spreadsheet and analysed by the Improvement Team.

**Results:** 30 patient records assessed.

97% of patients had an initial assessment, and 95% of community patients and 100% of inpatients had a named lead practitioner.

93% of patients had a care and support plan. All inpatients (100%) had timetabled daily activities with documented evidence of participation.

90% of community patients had access to specialist behavioural support. However, only 55% of applicable community patients were supported to choose where and how they live.

100% of restrictive interventions had a documented review.

77% of patients were prescribed antipsychotics, with 100% receiving psychological support alongside medication. Among these, 65% had a multidisciplinary review (MDT) of their antipsychotic use, with 45% reviewed within 3 months of initiation and 70% having subsequent reviews every 6 months.

**Conclusion:** Most patients had initial assessment and a named lead practitioner with specialist beahviour support in the community. Some areas of improvement include review of PBS plans and more MDT work around antipsychotics and physical health reviews.

